# Application and prospects of ultrasound combined with immunotherapy in cancer treatment of intensive care

**DOI:** 10.3389/fimmu.2025.1670527

**Published:** 2025-11-27

**Authors:** Yaping Wang, Cong Liu, Riley Lyu, Yan Zhang

**Affiliations:** 1 Department of Surgical Intensive Care Unit, Yantaishan Hospital, Yantai, China; 2 Johns Hopkins University School of Medicine, Baltimore, MD, United States

**Keywords:** ultrasound, ultrasound-assisted immunotherapy, tumor microenvironment, tumor, immunity, immunotherapy

## Abstract

The tumor microenvironment (TME) plays a crucial role in tumor initiation, progression, and metastasis, and immunotherapy targeting the TME has received increasing attention. However, single-agent immunotherapy has certain limitations and often requires combination with other adjuvant strategies to enhance therapeutic efficacy. Among these, ultrasound has emerged as a promising adjunct to cancer immunotherapy. By modulating the TME, ultrasound combined with immunotherapy shows great potential in enhancing antitumor responses. This review summarizes the application of various ultrasound modalities in enhancing antitumor immunity, improving the efficacy of immunotherapy, and regulating the TME. Ultrasound can amplify the therapeutic effects of immunotherapy through multiple mechanisms, including thermal effects, mechanical effects, microbubble cavitation, and sonodynamic therapy. Thermal effects induced by high-intensity focused ultrasound (HIFU) can destroy tumor tissues, releasing tumor antigens and heat shock proteins, thereby activating systemic immune responses. Mechanical approaches such as histotripsy can liquefy tumors without thermal damage, preserving antigenic structures and enhancing immune responses within the TME. Ultrasound-mediated microbubble cavitation increases vascular permeability, facilitating the delivery of immune cells and immune checkpoint inhibitors into tumor tissues and enhancing signal transduction to convert “cold” tumors into immune-active “hot” tumors. Sonodynamic therapy generates reactive oxygen species under ultrasound stimulation, inducing immunogenic cell death and reshaping the TME. Furthermore, this review outlines the research progress of ultrasound-immunotherapy combinations in various cancers, including lung cancer, breast cancer, and melanoma, demonstrating superior efficacy compared to immunotherapy alone. Ultrasound not only enhances antitumor immune effects but also enables real-time monitoring of tumor progression and immune modulation within the TME. Finally, the review discusses current challenges and future prospects. By systematically summarizing the types of ultrasound-assisted immunotherapy, their mechanisms within the TME, and recent advances in clinical applications, this article aims to provide a theoretical foundation and technical reference for developing ultrasound-immunotherapy strategies targeting the TME.

## 1 Introduction

In recent years, immunotherapy for cancer patients has attracted increasing attention. Immune signal transduction at single-cell resolution has provided new insights for the combination of immunotherapy and ultrasound in tumor treatment. The tumor microenvironment (TME) plays a critical role in tumor initiation, progression, and metastasis, particularly the immune microenvironment, whose regulatory capacity directly influences the efficacy of immunotherapy (1). In recent years, with the widespread application of tumor immunotherapies such as immune checkpoint inhibitors (ICI), the survival of patients with advanced malignancies has been significantly prolonged (2). However, not all patients benefit from these therapies. Especially in the context of critical illness, some patients exhibit a markedly suppressed TME, resulting in poor responses to immunotherapy (3). For instance, in patients with advanced melanoma and lung cancer, the overall response rate to immunotherapy is only 20%–30%, and many “immune cold tumors” fail to mount effective immune responses due to a lack of infiltrating T cells (4). Therefore, enhancing tumor immunogenicity, improving response rates to immunotherapy, and modulating the TME—particularly achieving more efficient and individualized immune interventions in critically ill cancer patients—have become key research priorities in the field of tumor immunotherapy.

Medical ultrasound technology offers unique advantages in the diagnosis and treatment of tumors in critical care settings, including real-time imaging, the absence of ionizing radiation, and its potential for physical therapy applications (5). Recent studies have demonstrated that ultrasound can be applied not only in tumor imaging diagnosis but also in modulating the TME through thermal and mechanical effects (3). For instance, physical ablation techniques such as high-intensity focused ultrasound (HIFU) can directly kill tumor cells and release tumor-associated antigens, thereby transforming lesions into “*in situ* vaccines” that elicit systemic immune responses (6, 7). In addition, the cavitation effect generated by ultrasound in combination with microbubble contrast agents can significantly increase local vascular permeability, facilitating the infiltration of immunotherapeutic agents and effector immune cells into tumor tissues, thereby enhancing local drug concentration (8–10). Simultaneously, ultrasound can induce immunogenic cell death (ICD) and the release of inflammatory cytokines, activating antigen-presenting cells such as dendritic cells (DCs), and enhancing T cell infiltration and immune signal transduction within the TME (11, 12). Therefore, ultrasound holds promise as a powerful adjunct to immunotherapy, facilitating the conversion of “immune cold tumors” into “immune hot tumors,” thereby improving the overall efficacy of immunotherapy in critically ill cancer patients (13, 14). (Reviewer 2 Q1) However, ultrasound combined with immunotherapy still has certain limitations. First, in clinical practice, the selection of ultrasound parameters (such as frequency, power, and pulse mode) varies widely, with different standards across regions and subjective differences among clinicians, which affects the comparability of treatment outcomes. Second, many novel materials are still in the experimental stage and generally face issues such as poor stability, insufficient biocompatibility, and challenges in clinical translation (15). Furthermore, in the field of critical care medicine, ultrasound combined with immunotherapy lacks large-scale randomized controlled trials to validate its long-term efficacy and safety (16). Therefore, future efforts in critical care should focus on optimizing clinical trial design, establishing standardized protocols, and developing new materials to further advance ultrasound-based immunotherapy.

(Reviewer 2 Q2) In the Surgical Intensive Care Unit (SICU), tumor patients are often postoperative or present with severe complications, frequently accompanied by immunodeficiency. As a noninvasive and bedside-operable technique, ultrasound can not only monitor tumor progression in real time but also reduce the inconvenience of frequent transfers between hospital departments (17). Meanwhile, addressing the immunodeficiency of SICU patients, ultrasound ablation and cavitation technologies can minimize surgical trauma while improving the immune microenvironment and promoting postoperative recovery (18). Looking forward, with the continuous development of integrated imaging and therapy, ultrasound combined with immunotherapy is expected to become an important adjunctive treatment for critically ill patients in the SICU, playing a significant role in both preoperative assessment and postoperative recovery.

Based on the aforementioned background, this review systematically summarizes the current applications and research advances of ultrasound-assisted immunotherapy in tumors from the perspective of the TME. First, we introduce the types of ultrasound-assisted immunotherapy in cancer patients, the relationship between inflammation and signal transduction in the TME, and the underlying mechanisms. Subsequently, we focus on three representative malignancies—lung cancer, breast cancer, and melanoma—to explore the specific applications of ultrasound technology in TME-related immunotherapy and the associated research progress. We then conduct a comparative analysis of current technological approaches and academic viewpoints, such as the differences between thermal and mechanical ablation in eliciting immune responses, as well as emerging explorations of sonodynamic therapy in immune activation and immune signaling (13). In addition, we evaluate the current research gaps and key technical challenges in the field. Finally, drawing on the latest research related to inflammation and signal transduction within the TME, we discuss future prospects for integrated image-guided immunotherapy, artificial intelligence (AI), targeted microbubble carriers, and sonogenetics. In summary, this review systematically outlines the categories of ultrasound-assisted immunotherapy and its mechanisms of action within the TME—including cytokine expression, immune cell modulation, and immune signal transduction—and further explores its application and progress in various tumor types. (Reviewer 5 Q1) This review will introduce the types and mechanisms of ultrasound-assisted immunotherapy, as well as its applications in various tumors, aiming to provide a theoretical basis and technical reference for advancing image-guided immunotherapeutic strategies targeting the TME.

## 2 Types and mechanisms of ultrasound-assisted immunotherapy

### 2.1 Thermal effects of ultrasound and immune activation

**Figure 1:**
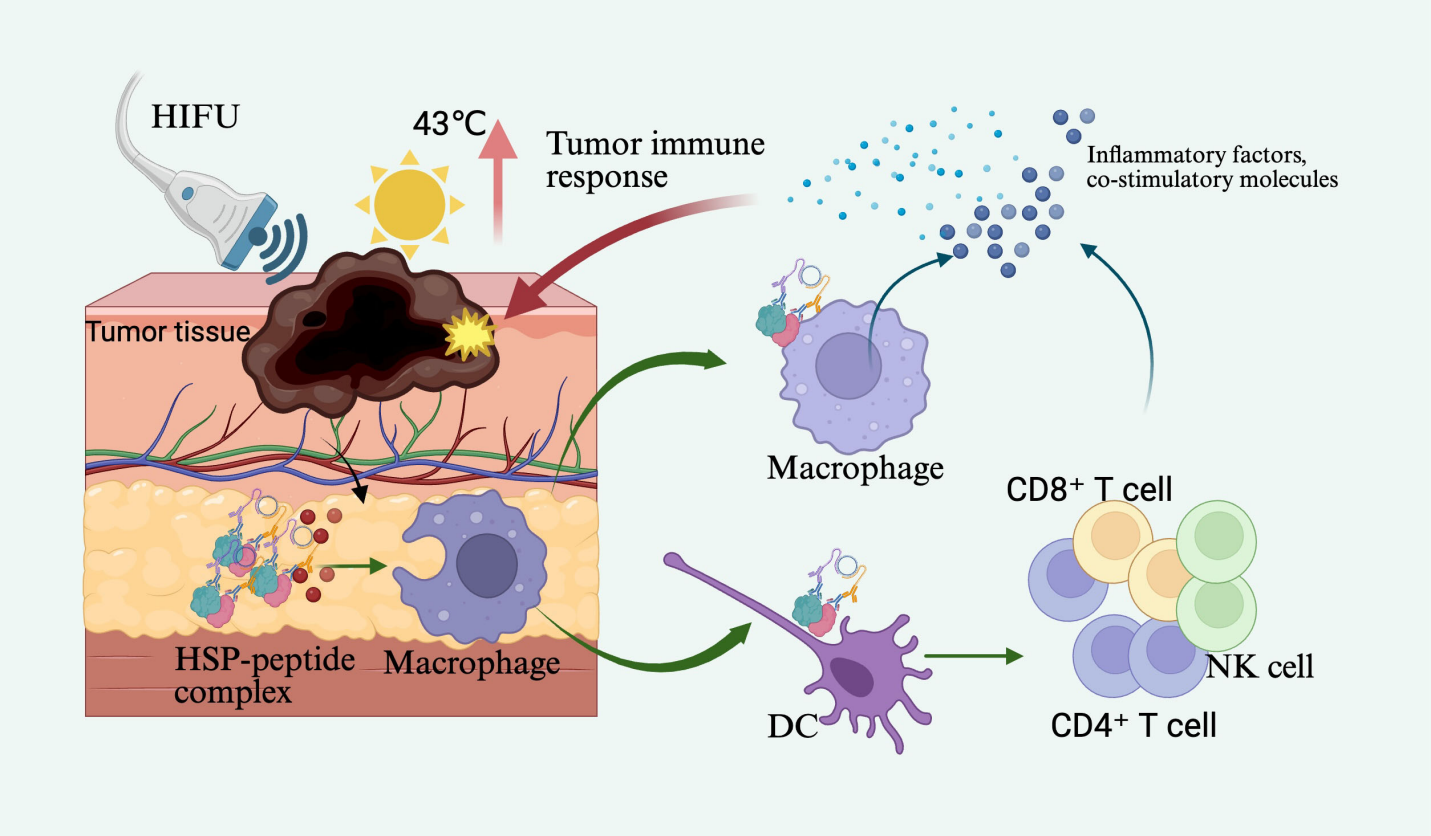
High-intensity focused ultrasound (HIFU) acts on tumor tissue, raising the local temperature to 43°C, thereby inducing the release of tumor-associated antigens and heat shock proteins (HSPs). These HSPs form HSP-peptide complexes, which are subsequently phagocytosed by macrophages or dendritic cells (DCs), leading to the expression of inflammatory cytokines and co-stimulatory molecules, ultimately activating tumor-specific adaptive immune responses.

In the treatment of critically ill cancer patients, HIFU can generate localized hyperthermia within tumor tissues, inducing coagulative necrosis or programmed apoptosis of tumor cells while simultaneously releasing a substantial quantity of tumor-associated antigens, heat shock proteins (HSPs), and other danger-associated molecular patterns (19–22). Studies have demonstrated that maintaining moderate thermal levels (~43°C) facilitates local immune cell infiltration, reduces stromal pressure, enhances membrane permeability, and upregulates HSP expression (19). HSPs can form complexes with antigenic peptides, which are taken up by macrophages and DCs, leading to the expression of pro-inflammatory cytokines and co-stimulatory molecules, thereby activating tumor-specific adaptive immune responses (19, 23, 24). In clinical management of critical oncology cases, evidence suggests that HIFU ablation significantly enhances antitumor immune activity in peripheral blood, maintaining stable levels of CD4^+^ and CD8^+^ T cells and natural killer (NK) cells. This immunological benefit surpasses the suppressive effects often associated with traditional surgical interventions, further supporting the role of thermal ablation as an effective immune adjuvant in immunotherapy (25–27). However, thermal ablation alone presents certain limitations. Sustained high temperatures may lead to extensive protein denaturation within tumor tissues, thereby reducing the immunogenicity of released antigens and compromising the efficacy of subsequent immune responses (28, 29). To address these limitations, non-thermal ultrasound ablation technologies, primarily based on mechanical disruption, have recently emerged. These approaches offer improved safety and precision, particularly suitable for critically ill cancer patients (**Figure 1**).

### 2.2 Mechanical effects of ultrasound and immunogenicity

The mechanical effects of ultrasound refer to tissue disruption primarily mediated by physical forces such as acoustic cavitation rather than thermal elevation. Representative technologies include emerging therapies such as histotripsy (10, 13, 30). Histotripsy utilizes high-pressure, low-duty cycle ultrasound pulses to induce rapid oscillation and collapse of cavitation microbubbles within the targeted tissue, mechanically “liquefying” tumor tissues into acellular debris (31, 32). Unlike thermal ablation, which often results in fibrotic scarring, histotripsy enables emulsification of tissue, promoting antigen exposure and presentation, and is more effective in eliciting immune responses (33). For example, Eric et al. (34) demonstrated in a murine melanoma model that following boiling histotripsy treatment of poorly infiltrated “cold” tumors, the level of tumor antigen in draining lymph nodes markedly increased within 24 hours—nearly tripling baseline levels—suggesting that this technique may help overcome immune resistance in tumors with low immunogenicity. Moreover, mechanical ablation preserves the native structure of tumor antigens by avoiding heat-induced denaturation, thereby facilitating effective dendritic cell activation and T-cell–mediated immune responses (10, 35, 36). In the context of critical oncology care, researchers have further compared the efficacy of mechanical ablation combined with immune checkpoint inhibitors (ICIs) versus ICIs alone, finding that the combination strategy significantly enhances systemic antitumor immunity and effectively suppresses distant tumor lesions (36, 37). Consequently, a key focus of current research is to optimize ultrasound parameters that favor mechanical effects while ensuring safety, in order to maximize the immunotherapeutic potential of ultrasound-mediated tissue ablation in critically ill cancer patients (32, 37, 38).

(Reviewer 5 Q3) Both mechanical and thermal effects are key mechanisms through which ultrasound acts on tumors, and they differ markedly in both their principles and duration. Mechanistically, the thermal effect primarily results from tissue absorption of ultrasonic energy and its conversion into heat, leading to local temperature elevation, protein denaturation, and the induction of ICD, whereas the mechanical effect arises mainly from acoustic pressure fluctuations and cavitation during ultrasound propagation, directly disrupting cell membranes, altering permeability, and rupturing lysosomal and mitochondrial membranes, ultimately inducing tumor cell death (22, 39). In terms of duration, the thermal effect typically exhibits a continuous, mild, and relatively stable energy release, while the mechanical effect features instantaneous and explosive energy output (40).

Moreover, under certain conditions, ultrasound-mediated mechanical and thermal effects can overlap. During prolonged ultrasound exposure, mechanical effects can induce relative motion between cells and the extracellular matrix, enhance cellular metabolism, cause microstructural damage, and increase ROS production, all of which may lead to localized temperature elevation (31). However, this heat generation is usually transient and localized, without causing widespread tissue heating. Notably, such localized thermal reactions induced by mechanical effects can further enhance ROS generation and drug release, thereby amplifying the overall antitumor efficacy of ultrasound therapy (41).

### 2.3 Ultrasound cavitation and microbubble-mediated immunomodulation

Ultrasound-induced cavitation refers to the oscillation, growth, and eventual collapse of microbubbles in a fluid under ultrasonic exposure, releasing high-energy physical forces (32, 42, 43). Due to the aberrant vascular architecture and elevated interstitial pressure in tumor tissues, passive drug penetration is often inefficient (44, 45). Ultrasound-targeted microbubble destruction (UTMD) utilizes microjetting and shock waves generated by cavitation to create transient and reversible pores and fissures in the vascular endothelium, thereby markedly enhancing the retention and permeability of macromolecular drugs within tumor regions (46, 47). (Reviewer 2 Q4) It is noteworthy that UTMD can act on vascular endothelial cells, enlarging the intercellular gaps and thereby facilitating the diffusion and penetration of drug molecules. However, this does not allow tumor cells to pass through these gaps into the bloodstream, preventing systemic dissemination. This is mainly because the diameter of the endothelial gaps induced by UTMD is limited, permitting only drug molecules to pass through, whereas the larger size of tumor cells prevents them from crossing the endothelial barrier into circulation (48, 49). Studies have demonstrated that UTMD not only physically disrupts tumor microvasculature and reduces local perfusion but also improves the uptake of immunotherapeutic agents within tumor tissues, particularly benefiting drug distribution in immunologically “cold” regions (4). For instance, Dong et al. (50) employed low-frequency ultrasound with microbubbles to transiently open the blood–brain barrier (BBB) in a murine glioma model, successfully delivering a CXCL10 chemokine and IL-2/anti–PD-L1 antibody complex into the tumor region. This approach enhanced CD8^+^ T-cell infiltration and cytotoxic activity. The phased delivery strategy, controlled via ultrasound frequency, effectively optimized immune cell activation and significantly improved the immunotherapeutic efficacy in brain tumors.

**Figure 2:**
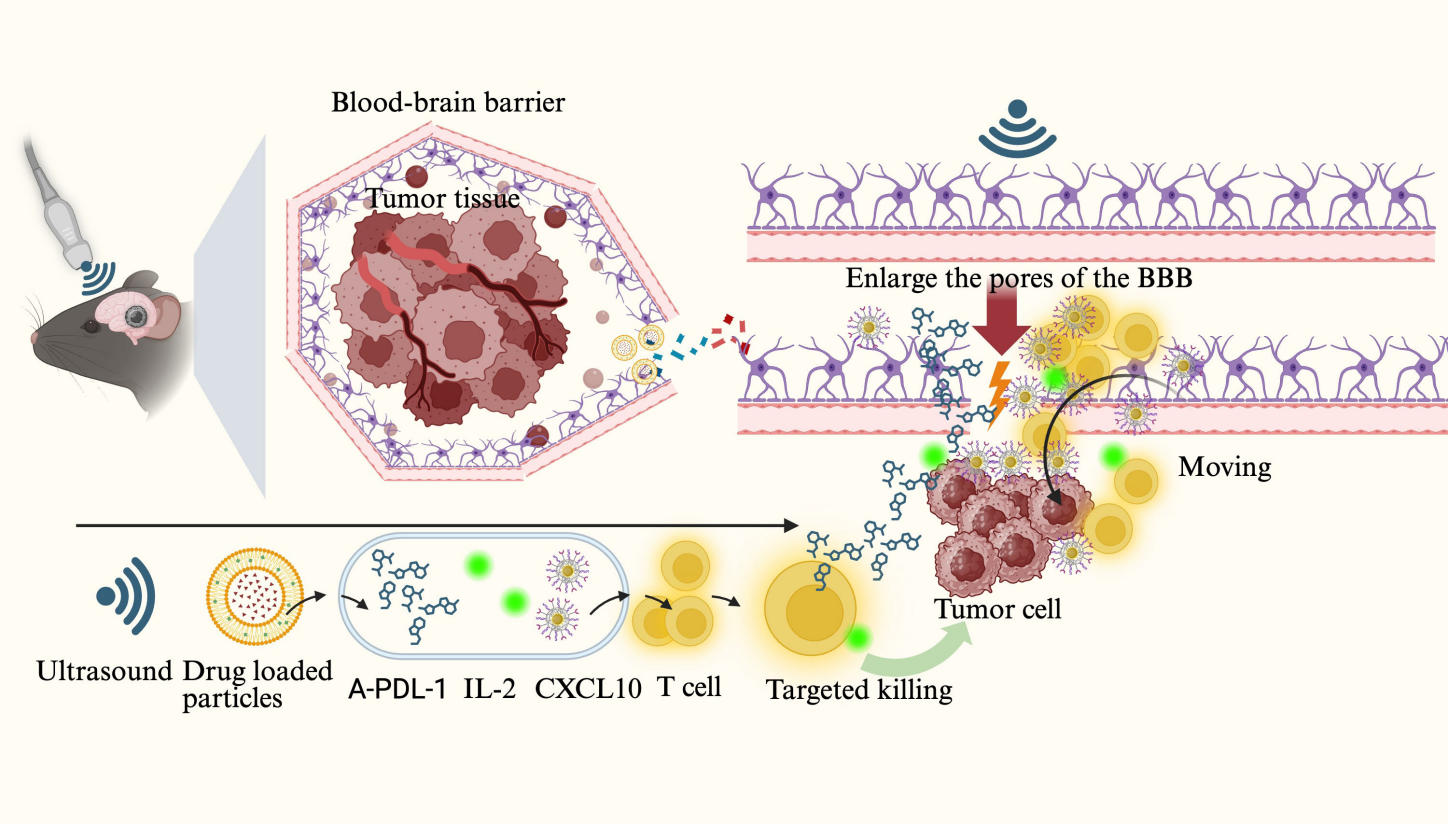
Ultrasound-induced microbubble cavitation acts on brain tumors, opening the tight junctions of the blood–brain barrier (BBB) while promoting the release of drug-loaded nanoparticles and the delivery of anti–PD-L1 antibodies, CXCL10 chemokines, IL-2, and other agents to form therapeutic complexes. These drugs and cytokines bind to the surface of T cells and diffuse through the expanded BBB gaps into the tumor region to eliminate tumor cells.

Another pivotal mechanism of cavitation lies in inducing localized tissue damage and cellular disruption, which subsequently releases tumor antigens and damage-associated molecular patterns (DAMPs), thereby activating immune cells and augmenting antitumor immune responses. Wu et al. (4), in a murine breast cancer model, demonstrated that low-intensity focused ultrasound–activated high-concentration microbubbles induced cavitation that effectively blocked intratumoral blood flow and directly lysed tumor cells, triggering ICD. Concurrently, the number of intratumoral DCs and cytotoxic T lymphocytes (CTLs) increased significantly, accompanied by elevated serum levels of immune mediators such as IL-12 and TNF-α. Notably, when combined with anti–PD-L1 therapy in this model, a synergistic enhancement in tumor suppression was observed, indicating that UTMD has the potential to amplify immune checkpoint blockade efficacy (51). In addition, nanoscale acoustically responsive carriers (e.g., nanobubbles, nanodroplets) exhibit similar immunostimulatory mechanisms. Due to their smaller size, these carriers can penetrate deeper into tumor cores via enhanced permeability and retention (EPR) effects, thereby further amplifying cavitation-mediated immune activation in poorly accessible regions (14). Collectively, ultrasound cavitation combined with microbubble technology demonstrates considerable potential in activating antitumor immunity through dual mechanisms—enhancing drug delivery efficiency and eliciting immune responses. This strategy offers promising applications in sensitizing tumors to immunotherapy and converting “immune-cold” tumors into immunologically active “hot” phenotypes (**Figure 2**).

### 2.4 Sonodynamic therapy and immunological effects

**Figure 3:**
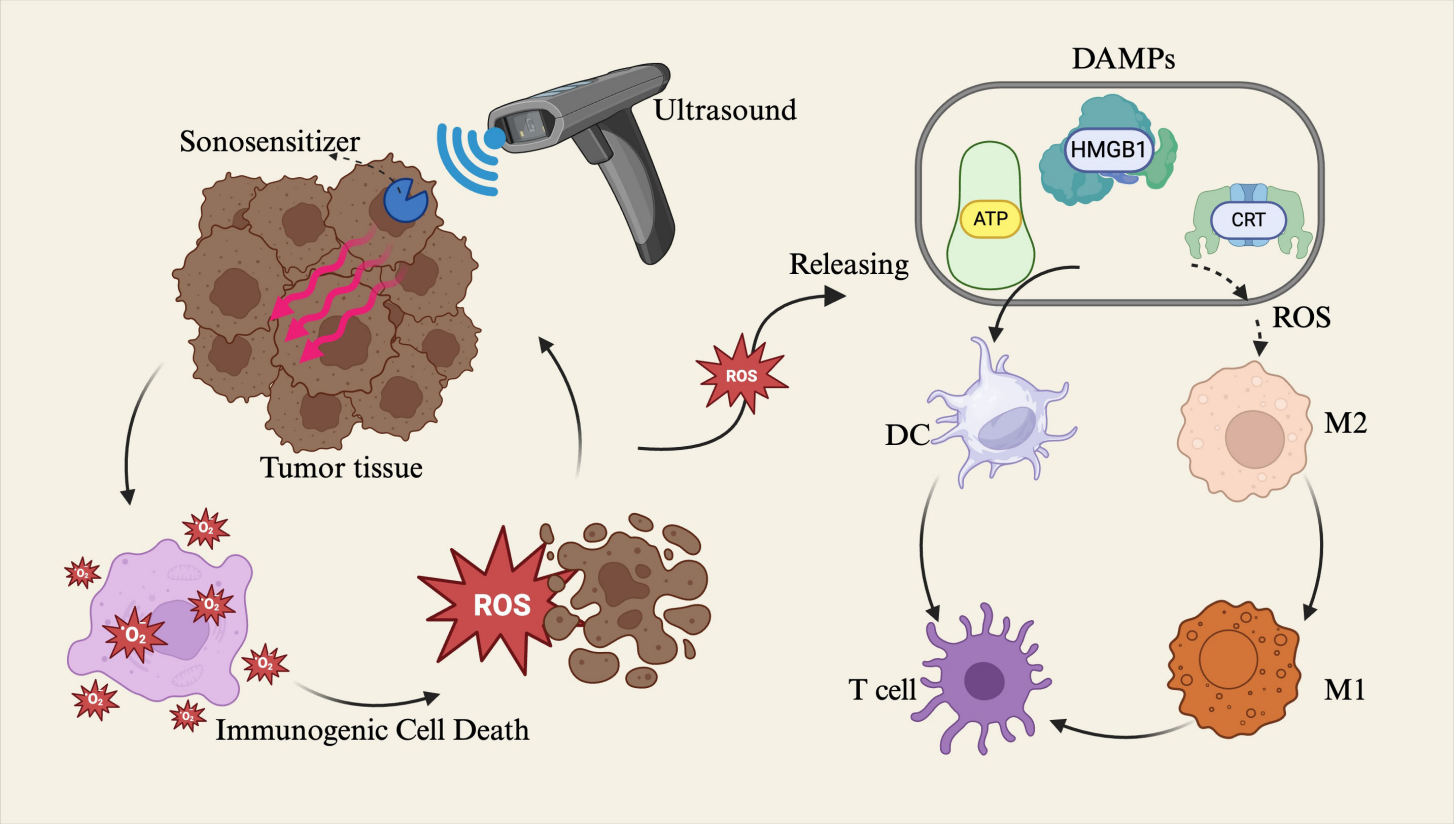
Sonodynamic therapy (SDT) applies ultrasound to tumor tissues, activating sonosensitizers that convert ultrasonic energy into reactive oxygen species (ROS). The generated ROS induce immunogenic cell death (ICD) in tumor cells and promote the release of damage-associated molecular patterns (DAMPs), including high mobility group box 1 (HMGB1), adenosine triphosphate (ATP), and calreticulin (CRT). These DAMPs facilitate the maturation of dendritic cells (DCs), thereby triggering T cell–mediated cytotoxic immune responses. Alternatively, ROS can modulate the tumor Microenvironment (TME) by promoting the polarization of macrophages from the M2 phenotype to the M1 phenotype, thus activating immune regulatory functions within the TME.

SDT is an emerging therapeutic strategy that employs ultrasound to activate sonosensitizers, generating large quantities of reactive oxygen species (ROS) to induce tumor cell death and stimulate antitumor immune responses (52, 53). Mechanistically similar to photodynamic therapy, SDT offers superior tissue penetration and focal targeting capabilities due to the physical properties of ultrasound. ROS generated during SDT can directly induce ICD in tumor cells and promote the release of DAMPs, including high mobility group box 1 protein (HMGB1), adenosine triphosphate (ATP), and calreticulin (CRT), thereby facilitating dendritic cell (DC) maturation and subsequent T cell–mediated cytotoxic immune responses (54). (Reviewer 5 Q2) ROS play a dual regulatory role in tumor initiation and progression. Low or sustained levels of ROS generally promote tumor formation and development, whereas high or acutely elevated levels of ROS exert significant antitumor effects (55, 56). Within the TME, moderate levels of ROS act as key signaling molecules that induce (ICD, thereby promoting the maturation and activation of antigen-presenting cells such as dendritic cells and enhancing antitumor immune responses. However, tumor cells often upregulate antioxidant factors—such as GSH—to resist oxidative damage and eliminate excessive ROS, maintaining a low ROS level conducive to their survival and proliferation, thus creating an immunosuppressive TME favorable for tumor growth (57). In contrast, ultrasound-mediated ROS release typically occurs as a short-term, high-intensity, and localized oxidative burst, capable of rapidly overcoming the tumor’s antioxidant defense barrier, disrupting the immunosuppressive microenvironment, and thereby achieving efficient tumor cell killing while enhancing the overall efficacy of immunotherapy (58, 59). Additionally, ROS can modulate the TME, for example by promoting the polarization of tumor-associated macrophages from the immunosuppressive M2 phenotype to the immunostimulatory M1 phenotype and enhancing antigen presentation, thus contributing to the reversal of local immune suppression (60–62). (Reviewer 3 Q4) In tumor therapy, inducing macrophages toward M1 polarization helps enhance antitumor effects. This is primarily because M1 macrophages can secrete various pro-inflammatory factors (e.g., TNF-α, IL-12) and generate ROS, which directly or indirectly kill tumor cells while activating the host immune system, thereby inhibiting tumor growth and metastasis (63, 64). However, excessive M1 polarization may trigger intense local immune-inflammatory responses. To address this, controlled-release strategies using biomaterials can finely regulate the degree of M1 polarization, thereby mitigating immune-mediated damage to surrounding normal tissues (65, 66). In recent years, advances in materials science have led to the development of various novel sonosensitizers, including organic agents such as porphyrins and phthalocyanines, inorganic nanomaterials such as metal oxides, and hybrid materials such as metal–organic frameworks (MOFs) (67, 68). For instance, a novel fluorinated covalent organic polymer sonosensitizer nanomaterial (PFCE@THPPpf-COPs) was developed to co-deliver perfluoroether, alleviating tumor hypoxia and enhancing SDT efficacy. This nanoplatform, when combined with anti-CD47 immunotherapy, significantly induced ICD, activated antitumor immune responses, enhanced T cell and M1 macrophage infiltration, and effectively suppressed tumor growth and recurrence (69). Another study encapsulated a sonosensitizer, the immune adjuvant R848, and tumor cell membranes within nanoparticles, enabling ultrasound-triggered ROS release and co-delivery of the adjuvant. This approach elicited potent systemic antitumor immune responses and immune memory effects, effectively eradicating both primary and metastatic lesions, thereby exhibiting a “vaccine-like” effect (70) (**Figure 3**).

Notably, in addition to apoptosis, ROS may also induce pyroptosis—a form of programmed cell death mediated by inflammasomes and characterized by the massive release of pro-inflammatory cytokines and DAMPs, thereby eliciting a more robust immune response (71, 72). Previous studies have demonstrated that optimizing the structure of sonosensitizers or simultaneously inhibiting tumor cell antioxidant defense mechanisms—such as depleting glutathione (GSH)—can enhance ROS-induced pyroptosis, thereby significantly improving the immune activation potential of SDT (73). Currently, sonodynamic immunotherapy still faces several challenges, primarily including the limited generation of ROS due to tumor hypoxia and the suboptimal selection and delivery efficiency of sonosensitizers (73). Nevertheless, with the rapid advancement of nanocarriers, composite materials, and targeted delivery systems, SDT is expected to become an important adjunctive strategy in immunotherapy, particularly offering promising clinical potential for overcoming the immunosuppressive microenvironment of solid tumors. (Reviewer 2 Q3) Ultrasound may overcome the immunosuppression of solid tumors primarily through the following mechanisms: on one hand, sonosensitizers and nanocarriers can generate ROS under ultrasound stimulation, inducing ICD and promoting antigen release, thereby remodeling the immune microenvironment and alleviating the immunosuppressive state of the TME (74). On the other hand, ultrasound-mediated cavitation can transiently increase tumor vascular permeability, facilitating the infiltration of immune cells and ICIs into the tumor core, thereby enhancing the efficacy of immunotherapy.

In summary, ultrasound significantly enhances the responsiveness of critically ill cancer patients to immunotherapy through multiple mechanisms, including thermal effects, mechanical disruption, cavitation, and sonodynamic therapy. (Reviewer 2 Q6) These effects can act independently or synergistically. On this basis, they can also activate the endogenous immune system, thereby achieving synergistic antitumor therapy (10). For example, the combined application of ultrasound thermal and mechanical effects in tumor treatment demonstrates significantly greater efficacy than either approach alone; when this method is combined with immune stimulation, it holds promise as a key strategy for eliciting systemic and durable antitumor immunity (37). These mechanisms not only facilitate antigen release and immune cell infiltration but also improve drug delivery efficiency and remodel the immune microenvironment—particularly demonstrating translational potential in “immune-cold tumors.”

(Reviewer 5 Q1) In conclusion, ultrasound technology provides strong physical support for tumor immunotherapy and effectively promotes the clinical implementation and development of precise treatment regimens for severe tumor cases. The following sections will further explore the specific applications and efficacy of these mechanisms in specific tumor types.

## 3 Application of ultrasound combined with immunotherapy in lung cancer

(Reviewer 1 Q1) In the SICU, managing critically ill patients, especially those with advanced malignancies, poses significant challenges (75). Ultrasound combined with immunotherapy plays an important role in their postoperative recovery and tumor progression monitoring. This therapeutic approach not only helps assess changes in postoperative tumor burden but also modulates the TME, thereby enhancing the efficacy of immunotherapy (47). In the field of critical care, common tumor types include lung cancer and breast cancer; these critically ill cancer patients often experience rapid disease progression and poor prognosis, making ultrasound combined with immunotherapy highly valuable for improving patient outcomes.

Advanced-stage lung cancer, particularly non-small cell lung cancer (NSCLC), is one of the primary indications for ICI therapy. However, therapeutic efficacy is often limited by tumor heterogeneity and variations in immune microenvironments across metastatic sites (76–79). Among these, liver metastases—a common and prognostically unfavorable form of spread in lung cancer—exhibit poor responsiveness to immunotherapy due to the intrinsic immune tolerance of hepatic tissue (80). To overcome the immunosuppressive nature of such “immune-cold” metastatic lesions, clinical efforts have explored the use of localized ultrasound-based interventions to modulate the hepatic immune microenvironment. An ongoing phase II clinical trial is currently evaluating the efficacy of HIFU combined with PD-1 blockade in lung cancer patients with liver metastases, under ultrasound guidance (81). The therapeutic strategy involves HIFU ablation of liver metastases one week prior to the initiation of immunotherapy. This approach aims to induce tumor debulking and antigen release via localized ablation, thereby enhancing the subsequent systemic immune response to PD-1 inhibition. Concurrently, the study seeks to assess HIFU-induced immune alterations and identify potential immunological biomarkers. However, this trial remains under follow-up, and the final results are expected to be released in December 2026 (**Table 1**).

**Table 1:** Applications of ultrasound combined with immunotherapy in different tumor types.

Tumor type	Ultrasound modalities	Combined immunotherapy strategies	Effects and advantages
Lung Cancer	HIFU local ablation, EBUS-TBNA biopsy, CEUS for therapeutic monitoring, and FUS-assisted BBB permeability enhancement.	Combined with PD-1/PD-L1 inhibitors to enhance immune response against liver metastases; EBUS-TBNA + ICI for treatment evaluation.	Tumor reduction + antigen release, modulation of the immune microenvironment, sensitivity to immunotherapy ↑; evaluation of ICI efficacy.
Breast Cancer	HIFU/LIFU ablation, CEUS dynamic monitoring, and SWE-based prediction of immune response.	Neoadjuvant chemotherapy + CEUS assessment; SWE + ICI treatment.	Induction of ICD, remodeling of TME, AI-assisted SWE to predict immune response.
Melanoma	CEUS for early identification of immune response, FUS to enhance immune activation, and SDT to boost antitumor activity.	FUS + CD40 agonist + ICI to activate antitumor immunity; HDRT + LDRT + UTMD to enhance immune cell infiltration in the TME.	Efficacy prediction; local ablation enhances T cell activation and infiltration ↑; growth of untreated distant tumors ↓, survival ↑.
Hepatocellular Carcinoma	Ablation techniques such as HIFU, RFA, MWA, PEI, and CRA combined with immunotherapy.	HIFU + PD-1 inhibitor to induce ICD in HCC.	Activation of DCs and T cells, sensitivity to anti-PD-1 agents ↑; antitumor efficacy ↑, complete tumor ablation rate ↑, local recurrence rate ↓.
Pancreatic Cancer	SDT to activate T cell immunity and ultrasound-based “diagnosis + therapy” integration.	SDT + PD-L1 inhibitor to induce immune response against distant tumors.	“Diagnosis + therapy” integration; induction of adaptive immune response, growth of distant tumors ↓.
Glioma	MB-FUS to assist BBB penetration; SDT combined with PDT.	MB-FUS + ICI to facilitate BBB penetration and improve ICI delivery; SDT + PDT to induce ICD.	Overcoming BBB barrier to drug delivery, drug delivery efficiency in the CNS ↑; intratumoral ROS ↑, antitumor efficacy ↑.
Bladder Cancer	Ultrasound combined with MBs for targeted drug delivery.	Ultrasound + MBs to deliver gemcitabine targeted to tumor tissue.	Precise drug delivery, drug side effects ↓, tumor growth ↓.

↑ means up, ↓ means down.

On the other hand, for primary lung lesions, the application of ultrasound is limited due to the air-containing nature of pulmonary tissue, which hinders direct imaging via transthoracic ultrasound. Consequently, ultrasound in such cases is primarily employed for interventional diagnostics and monitoring of metastatic lesions. Among these, endobronchial ultrasound-guided transbronchial needle aspiration (EBUS-TBNA) has emerged as the gold standard technique for assessing hilar and mediastinal lymph node metastases and obtaining tissue specimens. Notably, specimens acquired via EBUS can be used not only for tumor staging but also for the evaluation of immune biomarkers such as PD-L1 expression, thereby providing critical guidance for immunotherapy decision-making. Thus, in the context of expanding immunotherapeutic applications, the clinical importance of EBUS is increasingly recognized: by integrating imaging navigation with molecular diagnostics, EBUS offers vital support for tailoring individualized immunotherapy strategies in patients with advanced lung cancer.

For distant metastatic lesions of lung cancer (e.g., supraclavicular lymph nodes, adrenal glands, liver), ultrasound imaging serves as an important tool for therapeutic response evaluation during immunotherapy (82–84). Given the challenges in imaging interpretation, such as pseudoprogression frequently observed with immunotherapy, multiparametric imaging modalities are required to improve diagnostic accuracy. Contrast-enhanced ultrasound (CEUS) offers real-time information on tumor perfusion and helps differentiate between true disease progression and transient enlargement caused by immune cell infiltration (85, 86). For instance, in the monitoring of liver metastases, CEUS is highly sensitive to changes in tumor blood flow: effective immunotherapy is typically indicated by reduced or absent enhancement in the lesion center, suggesting tumor necrosis; conversely, increased perfusion may raise concern for tumor progression or excessive inflammatory response. Although no dedicated studies have systematically evaluated CEUS for monitoring immunotherapy response in lung cancer, its proven utility in assessing antiangiogenic treatments, such as with tyrosine kinase inhibitors (TKIs), provides a valuable reference (87). Future advancements in CEUS may include the development of microbubbles targeted to PD-L1 or other immune-related molecules, enabling molecular visualization of immunological changes within lung cancer metastases (88). Similar explorations have been reported in other tumor models, such as the use of VEGFR2-targeted CEUS microbubbles to monitor immunotherapy-induced vascular changes in melanoma (89), an approach that could be extended to lung cancer-related research.

It is noteworthy that focused ultrasound (FUS)–mediated BBB opening combined with immunotherapy is emerging as a cutting-edge therapeutic strategy for the clinical challenge of brain metastases in critically ill patients with lung cancer (90, 91). Lung cancer is associated with a high incidence of brain metastases; however, the majority of systemic therapeutics fail to effectively penetrate the BBB, thereby limiting the distribution of immune effectors to intracranial lesions. (Reviewer 5 Q4) FUS can overcome this limitation and enhance tumor sensitivity to drugs through the following mechanisms: first, FUS can transiently open the tight junctions of the BBB via mechanical cavitation, allowing macromolecular agents such as immune checkpoint inhibitors (anti–PD-1/PD-L1 antibodies) and cell-based therapies (e.g., CAR-T cells) to cross the BBB, thereby significantly increasing the infiltration of immune effector cells into the central nervous system (92). Sabbag et al. (93) demonstrated that this technique enables safe drug delivery and induces significant tumor-suppressive effects in murine brain tumor models. Therefore, for lung cancer patients with brain metastases, FUS under image guidance holds the potential to enhance immunotherapeutic sensitivity and improve the treatment prognosis of central nervous system metastases. (Reviewer 5 Q4) It is worth noting that this BBB opening is reversible and safe, without causing permanent damage (94). Secondly, FUS can induce an increase in local ROS levels, thereby activating antigen presentation and inducing ICD, which promotes the transition of the TME from an immunosuppressive to an immune-activated state and enhances the sensitivity of tumors to immunotherapeutic agents (58). In addition, studies have shown that FUS can improve local tumor perfusion and oxygenation, thereby inhibiting hypoxia-induced overexpression of HIF-1α and the activation of its downstream drug resistance signaling pathways, such as the PI3K/AKT pathway (95). In summary, FUS suppresses tumor drug resistance and enhances the overall efficacy of immunotherapy through multiple mechanisms, including reversible regulation of BBB permeability, upregulation of ROS production, induction of ICD, and modulation of hypoxia-related signaling pathways.

Overall, in the context of critical care for patients with lung cancer, ultrasound functions as a “behind-the-scenes hero” by indirectly enhancing the efficacy and personalization of immunotherapy through tumor debulking via ablation, guiding diagnostic sampling, and dynamically monitoring metastatic lesions (36). Although its direct application to primary pulmonary lesions remains challenging due to the air-containing anatomy of lung tissue, ultrasound is gradually demonstrating significant potential in managing metastatic sites, modulating the immune microenvironment, and activating systemic immune responses (76, 96). This is particularly important in critically ill lung cancer patients, where achieving rapid diagnosis and effective treatment under safe conditions is paramount. In such complex clinical scenarios, the multifaceted value of ultrasound is especially prominent. With the advancement of clinical and translational research, ultrasound is expected to become an indispensable component of the comprehensive immunotherapeutic framework for lung cancer.

## 4 Ultrasound-assisted immunotherapy in breast cancer

Breast cancer, particularly triple-negative breast cancer (TNBC), exhibits a generally low response rate to immunotherapy due to the absence of specific molecular targets, prompting increasing interest in combination strategies to enhance immune efficacy (97, 98). (Reviewer 5 Q6) For example, Adams et al. found that in metastatic TNBC, the objective response rate to pembrolizumab monotherapy was 5.3% in previously treated patients and 21.4% in treatment-naïve, PD-L1–positive patients (99, 100). In recent years, ultrasound technology has been extensively applied in the diagnosis and treatment of breast cancer, including for biopsy guidance, tumor ablation, and dynamic assessment of neoadjuvant therapy responses, thereby establishing a solid foundation for its integration with immunotherapy (101). ICIs, such as PD-1/PD-L1 antibodies, have shown preliminary efficacy in breast cancer, especially in TNBC (102). For instance, a phase III clinical trial conducted by Schmid et al. (103) demonstrated that the combination of atezolizumab and paclitaxel significantly prolonged progression-free survival in PD-L1–positive TNBC patients. Although the overall sensitivity of breast cancer to immunotherapy remains limited, multiple combination strategies—such as chemotherapy, targeted therapies, and local physical interventions like ultrasound ablation—are being actively investigated to overcome the barriers of “immune cold tumors” and expand the clinical indications of immunotherapy (104, 105). In the comprehensive management of critically ill breast cancer patients, leveraging ultrasound for precise tumor localization and therapeutic monitoring, thereby enhancing the specificity and response rate of immunotherapeutic interventions, represents a critical research direction that warrants further exploration.

### 4.1 Ultrasound ablation and mechanical effects facilitate immune activation

Breast tumors are often located in superficial regions, making them well-suited for local physical therapies such as HIFU and radiofrequency ablation (RFA). Previous studies have demonstrated that ablation of breast tumors can induce the release of tumor-associated antigens and activate host immune responses (106). Particularly in TNBC models characterized by poor baseline immune infiltration, local thermal ablation or mechanical disruption holds the potential to convert “cold tumors” into “hot tumors,” thereby enhancing the responsiveness to immunotherapy (107, 108). Wu et al. (4) proposed a strategy combining low-intensity focused ultrasound with targeted microbubble destruction (LIFU-TMD), which was applied in combination with PD-L1 antibody in a 4T1 breast cancer model. The results showed that LIFU-TMD caused rupture of aberrant tumor vasculature and a sharp decrease in blood perfusion, creating a “starved” microenvironment favorable to immune cell infiltration while inducing ICD, such as CRT exposure. Immunological analysis revealed significantly increased levels of DCs and CD8^+^ T cells in both tumors and their draining lymph nodes in the treated group, accompanied by elevated levels of immune-promoting cytokines such as IL-12 and TNF-α, ultimately leading to marked tumor suppression. These findings suggest that ultrasound microbubble-mediated mechanical intervention may substantially enhance TNBC sensitivity to ICIs, offering a novel immunotherapeutic sensitization strategy for critically ill breast cancer patients. Clinically, preliminary studies have explored the use of HIFU in the treatment of advanced breast cancer. A recent review noted that HIFU combined with immunotherapy not only demonstrates favorable safety but also significantly improves peripheral immune status, including increased CTL proportions and decreased regulatory T cell (Treg) ratios (109, 110). These studies provide both theoretical foundations and preliminary evidence for the clinical application of “ultrasound ablation plus immunotherapy” strategies in the management of critically ill breast cancer patients.

### 4.2 Application of ultrasound imaging in efficacy monitoring and response prediction

Ultrasound examination serves as a routine tool for follow-up management in breast cancer patients, offering the advantages of real-time feedback and high repeatability. It is particularly suitable for dynamically monitoring changes in tumor volume and tissue characteristics (111). During immunotherapy, conventional B-mode ultrasound can be used in conjunction with MRI/CT to assess trends in tumor shrinkage or enlargement. Especially when dealing with immune-related radiological phenomena such as pseudoprogression, ultrasound provides high-frequency evaluations of echogenic properties and blood perfusion, aiding in the differentiation between true progression and transient increases due to inflammatory responses (111, 112). CEUS has been widely applied in assessing responses to neoadjuvant chemotherapy in breast cancer, and its potential in immunotherapy monitoring is gradually emerging (113, 114). In a comparative study by Liu et al. (115), CEUS and MRI were evaluated for their performance in determining ablation efficacy within three days post-microwave ablation in 26 breast cancer patients. The results showed that both modalities had high sensitivity and negative predictive value. While MRI exhibited slightly better specificity and overall accuracy, CEUS successfully detected residual tumors missed by MRI in some cases, indicating complementary diagnostic value. For critically ill breast cancer patients, CEUS—being non-invasive, cost-effective, and suitable for repeated examinations—offers a feasible alternative for monitoring immunotherapy efficacy, particularly in scenarios where MRI is contraindicated or not amenable to frequent follow-up.

Emerging evidence suggests that shear wave elastography (SWE) combined with immunologic response analysis is becoming a novel focus in tumor imaging (116). Tumor stiffness reflects stromal composition and immune cell infiltration; tumors with high fibrosis and abundant cancer-associated fibroblasts (CAFs) often present as immunologically “cold,” characterized by low T cell infiltration and poor response to immunotherapy. Voutouri et al. (117) conducted a study using multiple murine tumor models, including breast cancer, and demonstrated a significant inverse correlation between tumor shear modulus measured by SWE and tumor suppression following ICI therapy (e.g., PD-1 blockade). Tumors with higher baseline stiffness and poorer perfusion responded less effectively to treatment. Administration of matrix-softening agents such as Tranilast improved perfusion and enhanced immune efficacy, indicating that reducing tumor stiffness may potentiate immunotherapeutic response. Building upon these findings, researchers incorporated AI methodologies to automatically extract CAF-related features from SWE images to predict immunotherapy response in TNBC patients. A specific CAF subtype with a “wound-healing” signature was identified, highly enriched in TNBC and closely associated with increased stiffness and immunosuppression. Moreover, the team developed a deep learning model trained on both murine and clinical datasets to noninvasively estimate the abundance of this CAF subtype using SWE imaging alone (118). Tumors with high CAF levels exhibited poor responses to PD-1 monotherapy; however, when combined with fibroblast growth factor receptor (FGFR) inhibitors, immune tolerance was reversed. These findings highlight the potential of AI-assisted SWE as a predictive imaging tool for immunotherapy efficacy in breast cancer, offering noninvasive, personalized decision-making support for critically ill patients and facilitating the implementation of precision immunotherapeutic strategies.

Taken together, current research in the field of breast cancer suggests that ultrasound-assisted immunotherapy exerts its impact on two critical fronts. On the therapeutic level, ultrasound combined with immunotherapy enhances tumor immunogenicity via localized physical modulation, induces ICD, and remodels the TME, thereby helping to reverse the “immune-cold” tumor phenotype. On the diagnostic and monitoring level, ultrasound imaging technologies—such as CEUS and SWE—enable dynamic evaluation of treatment responses, prognostic prediction, and support for the development of personalized therapeutic strategies, particularly suitable for the precise management of critically ill breast cancer patients. Looking ahead, with the advancement of clinical trials investigating ultrasound-immunotherapy combinations (e.g., HIFU plus PD-1 blockade in breast cancer), and the deeper integration of AI in ultrasound image analysis, ultrasound is expected to achieve clinical translational breakthroughs in the immunotherapy of treatment-resistant breast cancers, especially TNBC.

## 5 Application of ultrasound combined with immunotherapy in melanoma

Melanoma is one of the earliest solid tumors to achieve breakthroughs in immunotherapy, with PD-1 and CTLA-4 inhibitors significantly prolonging survival in patients with advanced disease (119–121). However, approximately 40% to 60% of patients exhibit a lack of durable responses to combined ICI therapy, particularly in “T cell–excluded” immune-cold tumors, which continue to pose a major challenge in the treatment of critical-stage malignancies (122, 123). Given that melanoma predominantly occurs in the skin or other superficial sites, it is highly amenable to ultrasound imaging and intervention, offering promising prospects for integrated application in this domain (124).

(Reviewer 5 Q5) Although the diagnosis of malignant melanoma can rely on clinical ABCDE criteria and pathological biopsy, ultrasound still plays a crucial role in its evaluation. Specifically: (1) High-frequency ultrasound can detect melanoma thickness and tumor invasion depth, which is of great importance for preoperative assessment of resection margins, surgical risk stratification, and postoperative prognostic evaluation (124). (2) Ultrasound examination can identify early lymph node metastases and guide image-assisted biopsy, thereby improving diagnostic accuracy (125). (3) During immunotherapy, ultrasound enables dynamic monitoring of disease progression with advantages of noninvasiveness and repeatability, and can be used to assess treatment response, detect recurrence, and guide subsequent therapeutic strategies. In summary, ultrasound effectively compensates for the static limitations of the ABCDE criteria and pathological biopsy, providing significant advantages in melanoma diagnosis, preoperative assessment, postoperative follow-up, and disease monitoring. Therefore, ultrasound examination is both necessary and of high clinical value for patients with melanoma.

### 5.1 Role of ultrasound in monitoring immunotherapeutic efficacy in melanoma

During immunotherapy, melanoma patients require frequent assessment of lesion dynamics, including primary tumors and regional lymph node metastases. Ultrasound exhibits high sensitivity in detecting subcutaneous and superficial lymph node metastases, capable of identifying early lesions with diameters of only a few millimeters (126). CEUS further enhances the visualization of tumor perfusion and neovascularization, offering critical insights for early evaluation of immunotherapeutic response. In a study conducted by Heimer et al. (89) using a B16 murine melanoma model, VEGFR2-targeted microbubbles were employed for CEUS detection. The results revealed a marked reduction in tumor perfusion five days post-treatment in the immunotherapy group, along with significantly lower VEGFR2 signal intensity at the late molecular imaging stage compared to controls. Immunohistochemical analysis demonstrated expanded areas of tumor necrosis, increased TIL infiltration, and decreased vascular density and VEGFR2 expression in the treatment group. These findings suggest that CEUS quantitative parameters, such as perfusion AUC and targeted microbubble binding intensity, may serve as non-invasive imaging biomarkers capable of reflecting therapeutic efficacy earlier than volumetric changes. CEUS has thus proven effective in differentiating between immune responses and irreversible progression in melanoma. Therefore, for melanoma patients with ultrasound-visible visceral metastases such as those in the liver or lymph nodes, CEUS can provide essential real-time efficacy monitoring, particularly suited for the individualized evaluation of immunotherapy in critically ill patients (126–128).

### 5.2 Ultrasound-assisted local therapy enhances systemic immune response

For unresectable melanoma lesions, physical ablation and local therapy play a pivotal role in inducing systemic antitumor immunity, exemplified by the classical “abscopal effect” (129). As a non-invasive ablation modality, FUS has demonstrated the potential to enhance immune responses in preclinical studies of melanoma. On one hand, HIFU can directly ablate melanoma tissue, leading to massive tumor cell death and antigen release, functioning as an “*in situ* tumor vaccine” (130). Hoogenboom et al. (131) applied HIFU mechanical ablation to a murine melanoma model and evaluated tissue fragmentation and pathological features under varying pulse numbers. The results revealed that the method effectively fragmented melanoma tissue, with residual viable cells and microvasculature in the treatment zone, and the degree of fragmentation was associated with the number of pulses and tumor density. On the other hand, cavitation-based mechanical ablation has shown unique advantages in melanoma models. Emerging techniques such as histotripsy significantly enhance local immune activity in T cell–poor “cold” tumors (10, 132). More importantly, this process is accompanied by pronounced ICD, potentially overcoming melanoma’s intrinsic low immunogenicity. Singh et al. (133), using a murine B16F10 melanoma model, implemented local boiling histotripsy combined with intratumoral injection of CD40 agonistic antibody (HT40) alongside FUS and ICI therapy to evaluate antitumor efficacy in “immune-cold” tumors. The study found that HT40 markedly enhanced the cytotoxic function of CD8^+^ T cells, remodeled the TME, and synergized with ICI to suppress growth of untreated distant tumors and prolong survival. These findings indicate that FUS and its derivative technique, histotripsy, exhibit considerable potential in immune activation for melanoma. Not only can they directly disrupt tumor tissue, but they also induce ICD and enhance T cell infiltration, triggering systemic antitumor immunity. Their synergistic application with immunotherapy offers a novel strategy for converting “cold” melanoma and achieving abscopal effects.

### 5.3 Exploration of sonodynamic therapy and other emerging technologies in melanoma

Melanoma patients often exhibit poor responsiveness to chemotherapy and a high propensity for brain metastasis, necessitating novel therapeutic approaches to complement traditional regimens (119, 121). In recent years, the application of SDT in melanoma has garnered increasing attention (38). Zheng et al. (134) developed a thermosensitive chitosan hydrogel based on CuO_2_ nanoparticles and buthionine sulfoximine (BSO), designed to enhance chemo-sonodynamic therapy for melanoma. The study revealed that this hydrogel promoted oxygen generation through a Fenton-like reaction, elevated ROS levels, and induced ferroptosis, thereby significantly enhancing antitumor efficacy against melanoma while concurrently accelerating the healing of infected wounds. Furthermore, considering the prominent neovascularization and lymphatic metastasis commonly associated with melanoma, emerging studies have shown that high-dose radiotherapy (HDRT), targeted ultrasound contrast microbubbles against tumor-associated antigens or angiogenic markers, and other molecular imaging–guided therapies offer promising outcomes in local control of melanoma (135). For instance, Patel et al. (136) conducted a Phase II clinical trial to evaluate the efficacy of HDRT combined with low-dose radiotherapy (LDRT) in patients with metastatic tumors, including immunotherapy-resistant melanoma. The results demonstrated that HDRT+LDRT significantly increased T cell and NK cell infiltration within tumor sites, improved the objective response rate and local control of melanoma lesions, and exhibited favorable safety profiles. Although these cutting-edge approaches remain in early-stage research, they underscore the feasibility and potential of “ultrasound plus molecular targeting” strategies in the precision treatment of melanoma.

It is worth emphasizing that immune-related adverse events (irAEs) are relatively common among melanoma patients receiving immunotherapy, and effective monitoring and mitigation of these side effects are of critical importance (137, 138). Ultrasound offers unique value in this context. For instance, in assessing organ-specific inflammation such as thyroiditis or immune-mediated hepatitis, conventional B-mode ultrasound enables noninvasive detection of parenchymal structural alterations, providing essential reference data for clinical interventions (139, 140). (Reviewer 3 Q7) Han et al. (141) reported that UTMD can enhance antitumor immune responses against melanoma by improving the local TME, promoting antigen release, and facilitating immune cell infiltration. In addition, UTMD can increase the local tissue penetration of drugs and modulate the tumor immune environment, thereby achieving a synergistic antitumor effect between ultrasound and immunotherapy, resulting in a “1 + 1>2” therapeutic outcome. Moreover, some studies have proposed that UTMD may reduce the incidence of certain immunotherapy-associated adverse effects by modulating tumor vascular permeability and enhancing immune cell infiltration; however, this hypothesis requires further validation (141).

In summary, melanoma, as one of the most rapidly advancing solid tumors in the field of immunotherapy, is emerging as a critical experimental platform for ultrasound–immunotherapy combination strategies. From therapeutic monitoring—such as early detection of immune responses via CEUS molecular imaging—to local interventions like FUS or histotripsy for inducing systemic antitumor immunity, and to novel technologies including SDT and targeted microbubble therapy, various ultrasound-based approaches have demonstrated promising results in melanoma. As these strategies continue to progress toward clinical implementation, ultrasound-assisted immunotherapy holds great promise for improving long-term survival and prognosis in the management of refractory melanoma within critical care settings.

## 6 Ultrasound-assisted immunotherapy in other tumor types

Beyond lung cancer, breast cancer, and melanoma, the concept of ultrasound-assisted immunotherapy is gradually being explored in other solid tumors, demonstrating distinct therapeutic advantages and mechanistic features.

### 6.1 Hepatocellular carcinoma

HCC is the most common form of primary liver cancer and represents the fifth leading cause of cancer-related death and the third leading cause of all-cause mortality worldwide (142, 143). The response rate of HCC to monotherapy with ICIs remains limited, while physical ablation is one of the current standard local treatment modalities for HCC (144). In recent years, studies have attempted to combine HIFU or percutaneous RFA with immunotherapy for the comprehensive management of intermediate to advanced HCC (145, 146). Luo et al. (147) conducted a comparative analysis of RFA with several other ablation techniques for liver cancer, including microwave ablation (MWA), percutaneous ethanol injection (PEI), and cryoablation (CRA), to evaluate therapeutic efficacy and safety. The results demonstrated that MWA and CRA achieved similar overall outcomes to RFA but offered higher complete ablation rates and lower local recurrence for larger tumors. Additionally, PEI combined with RFA further enhanced therapeutic efficacy, although it was associated with increased complication risk, thereby reasonably suggesting that combining HIFU with RFA may reduce the incidence of treatment-related complications. Yang et al. (148) developed a mechanical high-intensity focused ultrasound (mHIFU) system enhanced with perfluorohexane nanodroplets (NDs-PFH) and applied it in an HCC model to lower the cavitation threshold and induce ICD. The results revealed that this strategy not only significantly inhibited the growth of primary and distant tumors but also enhanced antitumor immune responses by activating DCs and T cells, and synergistically improved the efficacy of immune checkpoint blockade (e.g., PD-1 inhibitors). In conclusion, combining local ablation with immunotherapy may provide a synergistic treatment strategy for patients with intermediate to advanced HCC, improving response rates and long-term survival benefits.

### 6.2 Pancreatic cancer

(Reviewer 1 Q3) Pancreatic cancer is considered one of the most challenging “immune-cold tumors” due to its poor response to ICIs, which is not only related to its highly dense stromal architecture and immunosuppressive TME (149–151), but also to its inherent biological characteristics, such as rapid tumor proliferation, early distant metastasis, and resistance to conventional chemotherapy. These factors collectively contribute to the limited curative outcomes and poor prognosis of advanced pancreatic cancer (152, 153). In the critical care management of pancreatic cancer, ultrasound technologies have primarily been applied in local ablation and enhanced drug delivery. A study conducted by Nesbitt et al. (154) employed a bilateral pancreatic cancer mouse model to evaluate whether microbubble-mediated SDT could induce adaptive immune responses, and further examined its synergistic effects with anti-PD-L1 therapy on untreated distant tumors. The results demonstrated that SDT combined with anti-PD-L1 significantly inhibited distal tumor growth and increased infiltration of CD4^+^ and CD8^+^ T cells, suggesting that this strategy could activate systemic immune responses and improve checkpoint inhibitor efficacy. In another investigation, Delaney et al. (155) developed ultrasound contrast agent microbubbles composed of poly(lactic acid) (PLA) and PEG-PLA shells, encapsulating gemcitabine for both therapy and imaging of pancreatic ductal adenocarcinoma (PDAC). The results showed that the microbubbles exhibited excellent imaging performance and some antitumor potential. In this system, ultrasound functioned both as a diagnostic modality and a therapeutic trigger, enabling a theranostic approach to tumor management. These findings indicate that although pancreatic cancer remains a formidable challenge for immunotherapy, ultrasound, as a multifunctional tool, is offering novel strategies to overcome immune resistance and achieve integrated diagnosis and treatment.

### 6.3 Glioma

Gliomas represent one of the most challenging types of neuro-oncology tumors, with poor responsiveness to immunotherapy primarily due to the presence of the BBB and the “immune-privileged” status of the brain (156, 157). In recent years, FUS combined with microbubble technology has entered the clinical trial stage, aiming to repeatedly and controllably open the BBB in glioma regions to facilitate the delivery and therapeutic efficacy of immunoactive agents such as anti-PD-1 antibodies and CAR-T cells. Arvanitis et al. (158) developed and validated a closed-loop controlled microbubble-enhanced FUS (MB-FUS) system in a murine glioblastoma model, combining this modality with PD-1 immune checkpoint inhibition. The study demonstrated that the system enhanced intratumoral delivery and immunostimulatory activity of anti-PD-1, significantly prolonged survival, and induced the formation of memory T cells, resulting in protective immunity upon tumor rechallenge. This line of investigation is rapidly advancing and may offer a novel combinatorial strategy for immunotherapy of critical central nervous system malignancies. Additionally, photodynamic and sonodynamic therapies (PDT/SDT) for glioma are under preliminary exploration. These approaches utilize sonosensitizers and ultrasound to generate ROS within tumor tissues, thereby eliciting immune responses and eliminating residual tumor cells (159). These emerging technologies hold promise for overcoming current therapeutic limitations and providing new hope for refractory glioma.

### 6.4 Urologic and gynecologic malignancies

Urologic and gynecologic malignancies also exhibit promising potential for the integration of ultrasound with immunotherapy. Renal cell carcinoma (RCC) is relatively responsive to immunotherapy; however, for some patients who are ineligible for surgery due to tumor burden or anatomical constraints, non-invasive ablative modalities such as HIFU can serve as a bridging therapy (160, 161). In a murine model of muscle-invasive bladder cancer (MIBC), ultrasound combined with microbubble-mediated targeted delivery of gemcitabine, alongside radiotherapy, was evaluated for therapeutic efficacy and toxicity. The results demonstrated that this approach effectively delayed tumor progression while significantly reducing acute intestinal toxicity commonly associated with conventional chemoradiotherapy, highlighting its potential to enhance the safety profile of MIBC treatment (162). In the field of gynecologic oncology, advanced cervical and ovarian cancers are entering the era of immunotherapy investigation, with ultrasound microbubble-assisted drug or gene delivery emerging as a novel adjunctive strategy. For instance, targeted ultrasound molecular imaging (USMI) of VEGFR2 has been shown to effectively evaluate early-stage cervical cancer (FIGO stage IA1/IA2), accurately distinguishing lesions <3 mm from normal tissue. The imaging signal correlated well with microvessel density, indicating the potential of USMI for early, noninvasive screening and laying the groundwork for subsequent integration with immunotherapy (163).

(Reviewer 2 Q5) Furthermore, in addition to the aforementioned common solid tumors, ultrasound combined with immunotherapy also plays a significant role in hematologic malignancies. Gonzalo et al. (164) reported that using endobronchial ultrasound with transbronchial needle aspiration (EBUS-TBNA) for newly diagnosed lymphoma demonstrated moderate sensitivity and very high specificity, with even higher sensitivity for detecting lymphoma relapse. Diagnostic efficiency could be further improved through rapid on-site evaluation, increased sample volume, and flow cytometry. As a minimally invasive approach, this technique provides a reliable diagnostic tool for patients with suspected mediastinal lymph nodes or masses. (Reviewer 2 Q3) Compared with hematologic malignancies, ultrasound combined with immunotherapy demonstrates greater advantages in solid tumors. First, ultrasound can clearly assess tumor volume, blood flow, and histological characteristics, which is challenging in hematologic malignancies. Second, ultrasound-mediated ablation and cavitation effects can directly act on solid tumors, promoting antigen release and immune cell infiltration, thereby improving the local immune microenvironment. Finally, ultrasound combined with nanoparticles offers higher precision and targeting in solid tumors (165). Therefore, from the perspective of ultrasound-assisted immunotherapy, its application in solid tumors appears more promising than in hematologic malignancies and holds potential as an important strategy to overcome immune tolerance.

Overall, the integration of ultrasound and immunotherapy across various solid tumors remains in the exploratory stage, yet research efforts are commonly focused on enhancing immune infiltration, promoting antigen release, and overcoming both structural and functional barriers. In the future, as the biological characteristics of different cancer types are more deeply elucidated, ultrasound technologies are expected to be precisely optimized according to tumor-specific features. For instance, in highly fibrotic tumors (e.g., pancreatic and biliary cancers), mechanical forces may be applied to disrupt the stromal barrier; in tumors with prominent anatomical barriers (e.g., gliomas and bladder cancer), localized cavitation may enable targeted drug delivery; and in tumors with potential for abscopal effects, ultrasound ablation could be used to trigger systemic immune responses. These strategies are anticipated to continually expand the clinical boundaries of ultrasound-mediated tumor immunotherapy and intervention.

## 7 Discussion

Ultrasound-mediated immunotherapy represents a multidisciplinary integration of acoustics, bioengineering, and immunology. Divergences in research focus and technical approaches among different investigative teams have led to several contentious directions. Overall, the current discrepancies in scientific perspectives and technological strategies can be summarized into the following major aspects (1) The debate between thermal and mechanical ablation: The conventional view holds that HIFU-based thermal ablation effectively reduces tumor burden and releases tumor-associated antigens, thereby exerting a certain degree of immune activation. However, recent studies have indicated that high-temperature ablation may denature antigenic structures, induce localized hypoxia, and exacerbate the immunosuppressive microenvironment, making it suboptimal from an immunoactivation standpoint (96). In contrast, mechanical ablation techniques such as histotripsy, which avoid thermal effects, better preserve the native conformation of tumor antigens and generate tissue fragments that are more readily internalized and processed by immune cells such as DCs, thus being considered more favorable for eliciting systemic immune responses (166). In summary, each modality has distinct advantages: thermal ablation is clinically mature and widely applied, especially suitable for severe patients with heavy tumor burden, whereas mechanical ablation shows superior immunogenic effects but still requires further standardization in terms of device parameters and operational protocols. Future research may focus on exploring “thermal-mechanical synergy” strategies, wherein moderate heating is first employed to improve tumor permeability and perfusion, followed by mechanical pulses to enhance antigen release, ultimately achieving complementary effects and optimized immune activation (2). Technical divergence in cavitation microbubble applications: Some studies emphasize the use of conventional ultrasound contrast agent microbubbles for UTMD, which leverage cavitation effects to induce local blood flow disruption, enhance drug delivery, and stimulate localized immune activation; these systems are user-friendly and amenable to clinical translation (4). Alternatively, other researchers focus on the development of functionalized or targeted microbubbles, and even acoustically responsive nanocarriers capable of delivering checkpoint inhibitors such as anti–PD-1/PD-L1 and anti–CTLA-4 antibodies (47). Although functional microbubbles offer higher targeting specificity and therapeutic efficacy, they are hindered by high production costs, poor stability, and translational barriers. There is currently no consensus on which approach is superior. A tiered strategy may be feasible, wherein conventional UTMD is prioritized for rapid intervention in patients with complex immune states or severe disease, while functional microbubbles are reserved for specific indications or precision medicine scenarios, contingent upon data from large-scale clinical trials (3). Positioning and future prospects of SDT: Some studies suggest that SDT may serve as an alternative modality for deep-seated or phototherapy-insensitive tumors (e.g., brain tumors, pancreatic cancer), given its potential to induce ICD and function as an immune-activating tool. Critics, however, point out the limitations of SDT, including tumor hypoxia, the scarcity of effective sonosensitizers, and the limited penetration depth of acoustic energy, all of which contribute to inconsistent therapeutic efficacy (167). Notably, advances in materials science are gradually addressing these bottlenecks, with the introduction of novel platforms such as oxygen-carrying nanoparticles and MOF-based sonosensitizers (46, 68). In the future, SDT may be utilized in combination with immunotherapy to control multifocal or disseminated lesions in critically ill patients who are unsuitable for ablation-based interventions. (Reviewer 1 Q2) Furthermore, it is important to note that improper application of ultrasound—such as inappropriate parameter settings, excessive energy, or prolonged exposure—may negatively affect tumor treatment. Therefore, the careful selection of ultrasound frequency and intensity, combined with prior clinical experience, is crucial for balancing the therapeutic effects of ultrasound in tumor management.

(Reviewer 1 Q4) Conventional treatments such as surgery, radiotherapy, chemotherapy, and targeted therapy are often limited in efficacy due to the immunosuppressive characteristics of tumors, drug resistance, and the influence of the TME (168). Addressing these challenges, ultrasound combined with immunotherapy demonstrates distinct advantages: on one hand, ultrasound can improve the local TME and inhibit tumor growth through thermal and mechanical effects (51); on the other hand, ultrasound combined with immunotherapy can enhance the infiltration and distribution of drugs or immune cells within the tumor, thereby improving the efficacy of immunotherapy (169). Therefore, ultrasound combined with immunotherapy not only exerts local effects on tumors such as lung cancer and breast cancer but also produces systemic synergistic effects, playing a crucial role in enhancing overall therapeutic outcomes. (Reviewer 3 Q2) Compared with other imaging modalities, ultrasound offers several advantages over techniques such as radiofrequency ablation, Gamma Knife, cryoablation, or image-guided interventions like TACE. It enables real-time imaging, is noninvasive or minimally invasive, and provides high soft-tissue resolution. During examinations, ultrasound allows clear visualization of tumor morphology and surrounding tissue changes, which is important for assessing local tumor structures (170, 171). Moreover, ultrasound is not only a diagnostic tool but can also be used therapeutically, directly acting on tumor tissue through thermal and mechanical effects, modulating the local TME, enhancing immunotherapy efficacy, and promoting drug diffusion by increasing endothelial cell gap permeability to improve antitumor effects (48, 49). However, ultrasound has limitations, including limited penetration depth, lower energy concentration, operator-dependent variability, and certain constraints in treating deep-seated tumors (172, 173).

Overall, the current technological approaches in ultrasound-mediated immunotherapy are not mutually exclusive; rather, they each demonstrate distinct advantages depending on the biological characteristics of different tumors and specific clinical application scenarios. (Reviewer 1 Q5) In the field of critical care medicine, ultrasound combined with immunotherapy demonstrates significant clinical potential in critically ill cancer patients, particularly those with complex immune status. Ultrasound can noninvasively modulate the TME locally, enhancing drug penetration and immune cell infiltration (18). Moreover, as a noninvasive technique, ultrasound allows real-time monitoring of tumor progression, facilitating personalized treatment strategies for critically ill cancer patients (17). This approach helps improve the overall efficacy of immunotherapy and plays an important role in postoperative recovery. Future research should focus on delineating the appropriate indications for each technique while exploring the synergistic potential of their combinatory use. For patients undergoing cancer immunotherapy, ultrasound may emerge as a powerful tool for personalized intervention, facilitating the advancement of tumor treatment toward a new era of precision and efficiency.

Nevertheless, this field still faces notable research gaps and technical bottlenecks. Although ultrasound-immunotherapy strategies show promising potential, their clinical translation is challenged by multiple factors, including gaps in fundamental mechanistic understanding, limitations in material development, the need for robust clinical validation, and the establishment of comprehensive regulatory frameworks.

First, at the mechanistic level, numerous uncertainties remain. For example, what are the key mediators of immune responses induced by ultrasound? The mechanisms by which different ultrasound parameters—such as frequency, power, and duty cycle—affect immune activation are still poorly understood (37). Currently, no unified theory exists to guide the precise optimization of ultrasound settings specifically for enhancing immune responses, and the lack of standardized parameterization contributes to significant variability in results across studies (3). Establishing a standardized ultrasound immunotherapy parameter system is an urgent task in basic research and requires extensive validation through animal models and acoustic field simulations. Second, the development of sonosensitizers and nanocarrier materials remains at an early stage. Most current sonosensitizers are derived from photosensitizer structures and exhibit limitations such as high hydrophobicity, poor targeting, and low biocompatibility (174). Meanwhile, the design and scalable production of targeted microbubbles are also constrained. Future efforts should focus on developing “sonoimmunotherapeutic nanomedicines” that possess intelligent responsiveness and immune-targeting capabilities, such as nanoparticles or multifunctional microbubble systems capable of precise localization and immune activation. These advances will impose higher demands on materials science, pharmaceutics, and tumor immunology (175). Third, insufficient clinical validation remains a major bottleneck for broader implementation. Most current evidence is derived from small animal xenograft models, while clinical studies are still in early exploratory stages. The immune status and tumor heterogeneity in human patients, especially those in critical care settings, are far more complex than in animal models, limiting the translational applicability of preclinical findings. Finally, regulatory oversight and equipment compatibility also require concurrent improvement. Ultrasound-immunotherapy spans both medical imaging and oncology, and traditional departmental divisions may impede technological integration and blur responsibility boundaries. In response to the comprehensive management needs of critically ill cancer patients, it is essential to establish interdisciplinary collaborative mechanisms in clinical practice at an early stage, involving oncology, radiology, intensive care, pharmacy, and engineering teams (176).

In summary, the clinical translation of ultrasound immunotherapy hinges on resolving mechanistic uncertainties, overcoming material limitations, expanding clinical validation, and establishing a comprehensive interdisciplinary platform and technological ecosystem. For patients with complex tumors and those in critical care settings, this strategy holds promise as a pivotal enabling technology for future precision immunotherapy.

Looking ahead, we anticipate that the integration of ultrasound and immunotherapy in critically ill oncology patients will gradually progress from experimental exploration to clinical application, with several key trends warranting close attention (1) Imaging-immunotherapy integration: With the advancement of precision medicine, tumor treatment increasingly emphasizes real-time imaging surveillance and individualized feedback modulation (177). Ultrasound is expected to evolve into a therapeutic platform integrating both diagnosis and intervention. For instance, during ultrasound ablation or UTMD, real-time ultrasound imaging can monitor changes in tumor perfusion, tissue stiffness, and lesion margins; post-treatment, CEUS can noninvasively assess local immune responses and TME changes, thus aiding efficacy evaluation and refinement of immunotherapeutic regimens (3). This closed-loop model integrating imaging and therapy will improve decision-making accuracy, especially for dynamic monitoring and individualized immune interventions in critically ill patients with solid tumors (2). AI-assisted systems: AI technology will serve as a major driver in ultrasound-immunotherapy convergence by significantly enhancing image processing and clinical decision-making efficiency. (Reviewer 2 Q7) In recent years, artificial intelligence (AI) has rapidly advanced and, in combination with medical imaging technologies such as ultrasound, has achieved significant progress in disease diagnosis and treatment. Studies have shown that AI can extract features from baseline ultrasound images, such as elastography, to predict immune phenotypes and treatment responses in TNBC patients (118). (Reviewer 2 Q7) The study by Wang et al. (178) demonstrated that AI-assisted ultrasound imaging exhibited high sensitivity (0.88), specificity (0.75), and area under the curve (AUC 0.89) in predicting lymph node metastasis in breast cancer, significantly outperforming conventional non-AI ultrasound imaging. Similarly, Ji et al. (179) showed that deep learning–based models analyzing EBUS images, when combined with regions of interest, lymph node size on CT, and PET-CT results, significantly improved the diagnostic accuracy for mediastinal lymph node metastasis. Notably, the integration of PET-CT data yielded the most substantial improvement in model performance, highlighting the potential of AI-assisted ultrasound for precise detection in lung cancer. Future development may include AI-driven decision support systems that incorporate ultrasound imaging and clinical parameters to recommend ultrasound treatment settings, immunotherapy strategies, and potential efficacy assessments (180). Such systems could provide intelligent therapeutic planning for clinicians, particularly in screening and managing patients with high immune resistance or heavy tumor burden, thereby improving therapeutic efficacy and safety (181) (3). Targeted microbubbles and sonogenetics: Targeted microbubble technologies are advancing toward multifunctionality. Next-generation microbubbles may not only serve for molecular imaging but also act as carriers for delivering CRISPR-Cas9 gene editing tools or mRNA vaccines to tumor sites, enabling ultrasound-triggered, site-specific release and precise regulation (182). Additionally, sonogenetics—a burgeoning interdisciplinary field—is expanding ultrasound’s capacity to modulate cellular function. This approach leverages genetic engineering to express mechanosensitive receptors [e.g., Piezo channels (183)] in specific cells such as T or NK cells, allowing FUS to remotely activate cytotoxic functions (184). This strategy holds particular promise in critical care settings by achieving spatiotemporal control of immune cell activation and reducing systemic toxicity and adverse effects. While most sonogenetics research currently focuses on neuromodulation, its principles are fully extensible to immunotherapy and may offer revolutionary advances in future cell-based therapies (159).

## 8 Conclusion

In conclusion, this review systematically summarizes the research progress and clinical potential of ultrasound combined with immunotherapy across various tumor types. Through mechanisms such as thermal effects, mechanical forces, cavitation, and sonodynamic therapy, ultrasound modulates the TME and significantly enhances antitumor immune responses. When integrated with imaging-based monitoring, targeted delivery, and AI-assisted technologies, ultrasound-immunotherapy is gradually demonstrating its advantages of personalization, precision, and non-invasiveness in the treatment of cancers including lung cancer, breast cancer, and melanoma. Compared to monotherapy with ICIs, ultrasound-guided immunotherapy allows real-time monitoring of tumor progression and immune modulation at the TME level, thereby amplifying therapeutic efficacy. However, standardized treatment parameters and large-scale clinical validation remain lacking. Future directions will focus on the establishment of standardized protocols, extensive clinical trials, and the integration of emerging technologies such as AI to further advance the application of ultrasound-assisted immunotherapy in oncology.

## Glossary

ICIs: immune checkpoint inhibitors
TME: tumor microenvironment
HIFU: high-intensity focused ultrasound
ICD: immunogenic cell death
DCs: dendritic cells
AI: artificial intelligence
EBUS: endobronchial ultrasound
HSPs: heat shock proteins
NK: natural killer
UTMD: ultrasound-targeted microbubble destruction
BBB: blood–brain barrier
DAMPs: damage-associated molecular patterns
CTLs: cytotoxic T lymphocytes
EPR: enhanced permeability and retention
ROS: reactive oxygen species
SDT: sonodynamic therapy
HMGB1: high mobility group box 1 protein
ATP: adenosine triphosphate
CRT: calreticulin
MOFs: metal–organic frameworks
GSH: glutathione
NSCLC: non-small cell lung cancer
EBUS-TBNA: endobronchial ultrasound-guided transbronchial needle aspiration
CEUS: Contrast-enhanced ultrasound
FUS: focused ultrasound
TNBC: triple-negative breast cancer
RFA: radiofrequency ablation
LIFU-TMD: low-intensity focused ultrasound with targeted microbubble destruction
Treg: regulatory T cell
SWE: shear wave elastography
CAFs: cancer-associated fibroblasts
FGFR: fibroblast growth factor receptor
BSO: buthionine sulfoximine
HDRT: high-dose radiotherapy
LDRT: low-dose radiotherapy
irAEs: immune-related adverse events
HCC: Hepatocellular Carcinoma
MWA: microwave ablation
PEI: percutaneous ethanol injection
CRA: cryoablation
NDs-PFH: perfluorohexane nanodroplets
PLA: lactic acid
PDAC: pancreatic ductal adenocarcinoma
MB-FUS: microbubble-enhanced FUS
PDT/SDT: photodynamic and sonodynamic therapies
RCC: Renal cell carcinoma
MIBC: muscle-invasive bladder cancer
USMI: ultrasound molecular imaging.
